# HnRNPL inhibits the osteogenic differentiation of PDLCs stimulated by SrCl_2_ through repressing Setd2

**DOI:** 10.1111/jcmm.14166

**Published:** 2019-02-12

**Authors:** Xiaoshi Jia, Richard J. Miron, Chengcheng Yin, Hudi Xu, Tao Luo, Jiwei Wang, Rong Jia, Min Wu, Yufeng Zhang, Yuhong Li

**Affiliations:** ^1^ The State Key Laboratory Breeding Base of Basic Science of Stomatology (Hubei‐MOST) & Key Laboratory of Oral Biomedicine Ministry of Education Wuhan University Wuhan People's Republic of China; ^2^ Department of Periodontology, Cell Therapy Institute, College of Dental Medicine Nova Southeastern University Fort Lauderdale Florida; ^3^ Key Laboratory of Oral Medicine Guangzhou Institute of Oral Disease, Stomatology Hospital of Guangzhou Medical University Guangzhou China; ^4^ Hubei Key Laboratory of Cell Homeostasis, Hubei Key Laboratory of Developmentally Originated Disease, Department of Biochemistry and Molecular Biology, College of Life Sciences Wuhan University Wuhan China

**Keywords:** bioengineering, hnRNPL, osteoporosis, periodontal ligament stem cells, RNA splicing

## Abstract

Osteoporosis has been shown to intensify bone loss caused by periodontitis and both share common risk factors. One strategy utilized to manage the disease has been via the release of Sr ions by Strontium Ranelate having a direct effect on preventing osteoclast activation and promoting osteoblast differentiation. Previously we have developed and characterized porous Sr‐mesoporous bioactive glass (Sr‐MBG) scaffolds and demonstrated their ability to promote periodontal regeneration when compared to MBG alone. Our group further discovered a splicing factor, heterogeneous nuclear ribonucleoprotein L (hnRNPL), was drastically down‐regulated in periodontal ligament stem cells (PDLCs) stimulated by Sr through the activation of AKT pathway. Furthermore, hnRNPL restrained the osteogenic differentiation of PDLCs through down‐regulating H3K36me3‐specific methyltransferase Setd2. The goal of the present study was to investigate the mechanism of periodontal regeneration stimulated by Sr It was first found that the epigenetic mechanism of splicing factor hnRNPL participated in the osteogenesis processing of PDLCs stimulated by SrCl_2_. Meanwhile, the different role of hnRNPL and SET domain containing 2 (Setd2) may provide some implication of the treatment of periodontitis patients simultaneously suffering from osteoporosis.

## 1 INTRODUCTION

Periodontal bone defects is one of the most important part of maxillofacial bone defects.^1^ During the repair of periodontal bone defects, progenitor cells can be recruited into the defect area and then differentiate into periodontal ligament cells which have multi‐lineage differentiation potential. However, spontaneous periodontal bone regeneration is always difficult to achieve since the rate of proliferation epithelial cells and fibrocytes overwhelm the differentiation of periodontal ligament stem cells (PDLCs).^2^ Moreover, bone loss caused by periodontitis is accentuated in osteoporotic patients and the treatment is deemed significantly more complicated,^3^ Therefore, effective and precision therapies are constantly sought as treatment options for the continuously rising number of patients with both periodontal disease and osteoporosis.

A variety of treatment options for periodontal bone defects healing have been proposed using novel tissue engineering scaffolds.^4^ In recent years, mesoporous bioactive glass (MBG) has been utilized as a bioactive material with pore sizes ranging from 5 to 20 nm and a well‐ordered mesoporous channel structure capable of promoting new bone formation in vivo.^5^, ^6^, ^7^, ^8^ Strontium Ranelate has been applied clinically to prevent osteoporosis and strontium has been shown to activate calcium sensing receptor (CaSR) and downstream protein phosphorylation to promote osteogenesis.^9^ Recently our group developed a kind of MBG scaffold containing strontium and demonstrates its positive effective on promoting osteogenesis and periodontal regeneration in osteoporotic animal models.^10^, ^11^, ^12^ Although strontium ions play an important role by inhibiting bone resorption and promoting bone formation through different signaling pathways,^13^, ^14^ the mechanism in the nucleus at the genetic level during periodontal regeneration stimulated by Sr ions remains unclear.

Alternative splicing is a kind of the regulatory mechanism in eukaryotic cells, whereby the pre‐mRNA can be spliced into multiple mature mRNA due to the introns existing in the DNA, followed by translation to produce a variety of proteins.^15^, ^16^ HnRNPL is an important member in the family of ribonucleic protein splicing factors enriched in cell nucleus and plays an important role in many physiological and pathological processes.^17^, ^18^ HnRNPL contains four RRM domains, a proline‐rich sequence separating RRMs 2 and 3 and a glycine‐rich N‐terminal domain.^19^ This ubiquitous splicing‐regulatory protein is critical for the oncogenesis such as osteosarcoma and the development of T cells.^20^ It can also inhibit P53 to prevent DNA damage‐induced apoptosis in embryonic stem cells.^21^ Moreover, cluster analysis of genes regulated by hnRNPL showed genes containing hnRNPL‐regulated exons were associated with chromatin regulation, DNA modification and transcription, implied the epigenetic role in hnRNPL‐related splicing process.^22^ HnRNPL can interact with Set2 and is related to the retention of introns in eukaryotic cells.^22^ We have previously shown the positive regulation of Setd2 in the process of osteogenesis stimulated by Sr.^23^ However, whether hnRNPL can regulate the osteogenesis process is still lack of evidence.

In this study, we demonstrated that hnRNPL could play a role in the osteogenic differentiation of PDLCs stimulated by Sr by regulating Setd2. Following stimulation with Sr, the AKT pathway was activated and then repressed the expression of splicing factor hnRNPL, ultimately promoted the osteogenic differentiation of PDLCs through Setd2 up‐regulation.

## 2 MATERIALS AND METHODS

### 2.1 Preparation of MBG and Sr‐MBG scaffolds

Preparation and characterization of Sr‐MBG scaffolds were performed as previous described.^24^ Porous strontium‐incorporated mesopore‐bioglass (Sr‐MBG) scaffolds were prepared by incorporating different contents of Sr (molar: 5%) into MBG using co‐templates of nonionic block polymer P123 (EO20‐PO70‐EO20) and polyurethane sponges. In brief, to prepare MBG scaffolds containing 5% of Sr, 8 g of P123 (M_w_ = 5800, Aldrich), 12.5 g of tetraethyl orthosilicate (TEOS, 98%), 2.8 g of Ca(NO_3_)_2_.4H2O, 1.1 g of SrCl_2_ (Aldrich), 1.46 g of triethyl phosphate (TEP, 99.8%) and 2 g of 0.5 M HCl were dissolved in 120 g of ethanol (Sr/Ca/P/Si=5/15/5/75, molar ratio, named Sr‐MBG) and stirred at room temperature for 24 hours. The polyurethane sponges (20 ppi) were cleaned and completely immersed into this solution for 10 minutes, then transferred to a Petri dish to allow evaporation at room temperature for 12 hours. This procedure was repeated three times. Once the samples were completely dry, they were calcined at 700°C for 5 hours yielding the Sr‐MBG scaffolds. MBG scaffolds without Sr (Sr/Ca/P/Si=0/15/5/80, molar ratio, named MBG) were prepared by the same method except for their Sr and Si contents.

### 2.2 Animals and surgical procedures

Establishment of the osteoporotic rat models were performed as previous described^12^, ^24^, ^25^ in accordance with the policies of the Ethics Committee for Animal Research of Wuhan University, People's Republic of China, and approved by the Ethics Committee at the School of Dentistry, prior to the start of this experiment. All experimental procedures were approved by the Ethics Committee for Animal Use of the Institute of Biomedical Sciences, under protocol number 134/2012. 15 mature female Wistar rats (10 weeks old, mean body weight 230 g) were purchased and kept at 20‐25°C under a 12 hour‐light/dark cycle and allowed food and water ad libitum. All operations were carried out under sterile conditions with a gentle surgical technique. The surgeon was blinded to the treatment.

After one week for acclimatizing to the new laboratory surroundings, an osteoporosis animal model was carried out by bilateral ovariectomy (OVX) under sterile conditions with a minimally invasive surgical technique. Briefly, when general anesthesia was achieved, rats were operated with 10 mm linear bilateral lumbar lateral skin incisions. Then the enterocoelia was exposed by blunt dissection of muscle and peritoneum. The bilateral ovaries were removed gently following ligation of the ovarian artery and vein. Then the overlying muscles and epithelial tissues were sutured in multi‐layers. A single intramuscular dose of penicillin 40,000 IU/mL was then administered postoperatively. No significant perioperation or post‐operation fractures were produced.

Two months after the osteoporosis model was established, periodontal fenestration defects (standardized with 2.8 mm in length, 1.4 mm in height and 0.5 mm in depth) were created as previous described.^12^ Briefly, under general anesthesia, rats were subjected to bilateral extra‐oral incision at the base of the mandible. The buccal mandibular bone overlying the first molar roots was removed to create a defect (2.8 mm in length, 1.4 mm in height and <0.5 mm in depth) using a size‐4 round bur. The procedure was performed under an operating microscope to avoid perforation of intraoral mucosa. The roots of the first molar were carefully denuded of their periodontal ligament, overlying cementum, and superficial dentin. The height was standardized to the width of the round bur (diameter 1.4 mm) and extended longitudinally to either side. Then, two scaffolds were gently implanted respectively into the bilateral periodontal fenestration defects (MBG in the left and Sr‐MBG in the right defect). Following implantation of scaffolds, the muscle and the skin were repositioned and sutured separately. Postoperatively, penicillin (40,000 IU/mL, 1 mL/kg) was injected intramuscularly for 3 days. Four weeks after surgery, the rats were sacrificed by sodium pentobarbital, following a protocol approved by the Institutional Animal Care and Use Committee (IACUC).

### 2.3 Immuno‐histochemical staining

The immune‐histochemical staining was performed as previous described.^23^ Periodontal bone regeneration samples were immersed in 10% EDTA changed twice weekly for 3 weeks until decalcification, followed by a gradient dehydration for embedding in paraffin. To compare the bone regenerative properties of each scaffold, Masson staining was performed in accordance with the manufacturer's protocol (MXB biotechnologies, China). For immunohistochemistry staining, serial sections of 5 μm were cut and mounted on polylysine‐coated slides. Following the process of deparaffinazation, rehydration and washing with PBS, the sections were then incubated with 0.3% hydrogen peroxide for 20 minutes followed by incubation with BSA. The sections were thereafter incubated with the primary antibody for Runx2 (1:400; Abcam ab76956, Abcam, England), hnRNPL (1:100; SANTA CRUZ SC‐32317, Santa Cruz, CA), Setd2 (1:800; ABclonal A3194, ABclonal, USA), H3K36me3 (1:200, ABclonal A2366, ABclonal, USA) overnight at 4°C. In accordance with the manufacturer's protocol of immunohistochemical kit (MXB biotechnologies, China) and visualized by 3,3‐diaminobenzidine tetrahydrochloride (DAB) (Zhongshan Biotechnology, China). Lastly, the sections were counterstained with hematoxylin. Quantitative analysis of immunohistochemical staining was performed by determining the percentage of positive cells of all cells in the zones of the observed area. The percentage (*P*) of bone regeneration was calculated as: *P* = positive area/total defect area × 100%. Newly formed bone was identified by its different staining of eosin and morphological structure as previously described.^10^

### 2.4 Isolation and culture of human PDLCs

Human PDL cells were cultured in α‐MEM medium (Hyclone, Thermo Fisher Scientific Inc., New York, NY, USA) supplemented with 10% fetal bovine serum (FBS; Gibco, Life Technologies Corporation) and 100 U/mL penicillin G and 100 mg/mL streptomycin (HyClone) at 37°C with 5% CO_2_. Osteogenic inducing medium comprised α‐MEM containing 10% FBS, 100 U/mL penicillin and 100 mg/mL streptomycin, 10 nmol/L dexamethasone, 10 mmol/L β‐glycerophosphate, 50 μg/mL l‐ascorbic acid and different concentrations of SrCl2 (0, 0.01, 0.1, 1, 3 mmol/L). Primary PDLCs were seeded from passages 4‐6 at a density of 5000/well in 96‐well culture plates for cell proliferation experiments and 300 000/well in 6‐well culture plates for real‐time PCR and alizarin red experiments. For experiments lasting longer than 5 days, medium was replaced with fresh one twice weekly.

### 2.5 Viral transfection

Viral packaging and transfection was performed as previously described.^26^ The target sequences for shRNA1 were: hnRNPL of humans: 5′‐CGACAACCAAATATACATT‐3′; The target sequences for shRNA1 were: hnRNPL of humans: 5′‐CTTGAATGTGTTCAAGAAT‐3′; Setd2 of humans: 5′‐TAGTACACCAAGACTCCAG‐3′. pHAGE‐HA‐SETD2 plasmid was available from Dr Min Wu (Wuhan University). The overexpression plasmid or shRNA plasmid, as well as two helper vectors (pMD2G and pSPAX2) were transfected into HEK293T cells using Neofect transfection reagent. The viral supernatants were collected after 48 hours and then concentrated. The supernatants were then used to transfect PDLCs in the presence of 10 μg/mL polybrene (Sigma). Twenty‐four hours later, the viral supernatants were removed and cultured with fresh growth medium.

### 2.6 Alizarin red staining and quantification

Alizarin red staining was performed to determine the presence of extracellular matrix mineralization in vitro. PDLCs were fixed in 4% formaldehyde for 15 minutes after culture for 21 days and stained with 0.01% alizarin red solution (pH 4.2) at room temperature for 1 hours. For quantifying the relative amount of calcium, the stained nodes were dissolved using 200 μl of 1% cetylpyridinium chloride (Sigma, USA) per well at room temperature for 4 hours and then the OD value was measured at 560 nm.

### 2.7 ALP staining and quantification

PDLCs were fixed in 4% formaldehyde for 15 minutes after being cultured for 7, 14 and 21 days and stained with ALP staining kit (Beyotime Biotechnology, China) at 37°C for 30 minutes.

### 2.8 Protein extraction and western blotting

Protein extraction and western blotting was performed as previously described.^26^ PDLCs were starved for 24 hours, then stimulated by SrCl_2_, collected with PBS, mixed with loading buffer and then heated at 95°C for 10 minutes for total protein denaturation. Nuclear and cytoplasmic proteins were extracted by Nuclear and Cytoplasmic Protein Extraction Kit (Beyotime Biotechnology, China). The protein contents were determined by using a bicinchoninic acid (BCA) assay kit (Thermo Scientific).

Proteins were separated by SDS‐PAGE then transferred to nitrocellulose membranes. The membranes were blocked using 5% non‐fat milk or 5% BSA for 60 minutes at room temperature. Membranes were incubated with primary antibodies against hnRNPL (1:1000), Setd2 (1:1000), H3K36me3, P‐AKT (1:2000), AKT(1:1000), P‐CREB (1:2000) at 4°C overnight, followed by secondary antibodies for 1 hours at room temperature. Protein bands on the membranes were visualized using Western Bright ECL HRP substrate Kit (Advansta).

### 2.9 RNA extraction and RT‐qPCR

Total RNA was extracted with Trizol reagent (TriPure Isolation Reagent, Roche Applied Science, Germany) according to the manufacturer's instructions. The concentration and quality of the total RNA samples were measured using Nanodrop2000 (Thermo Scientific). Complementary DNA was synthesized from 1 μg of total RNA using PrimeScript RT‐PCR Kit (TaKaRa, Japan) following the manufacturer's protocol. RT‐qPCR was performed with PrimeScript RT‐PCR Kit (TaKaRa, Japan). The primers are shown in Table 1.

**Table 1:** The primer sequences used in RT‐qPCR

Gene	Organism	Primer Sequence
*hnRNPL*	Homo sapiens	Forward 5′‐CTGGAGCAGAGGCAGCA‐3′
		Reverse 5′‐GTTTTGTGCGGGTCATCGTAG‐3′
*Setd2*	Homo sapiens	Forward 5′‐TCTCCAAGCAACCAGCCATT‐3′
		Reverse 5′‐CCGCTTCACTCCCAGTTCAT‐3′
*ALPL*	Homo sapiens	Forward 5′‐CAACCTGAGCTGCCTTTCTCAG‐3′
		Reverse 5′‐AGAACTGTGTCCCGGCTTCTC‐3′
*RUNX2*	Homo sapiens	Forward 5′‐GCGCATTCCTCATCCCAGTA‐3′
		Reverse 5′‐GGCTCAGGTAGGAGGGGTAA‐3′
*BGLAP*	Homo sapiens	Forward 5′‐TCCTTTGGGGTTTGGCCTAC‐3′
		Reverse 5′‐CCAGCCTCCAGCACTGTTTA‐3′
*GAPDH*	Homo sapiens	Forward 5′‐TCAGCAATGCCTCCTGCAC‐3′
		Reverse 5′**‐**TCTGGGTGGCAGTGATGGC‐3′

### 2.10 Statistical analysis

GraphPad Prism software (GraphPad, San Diego, CA) was used for the statistical analyses. Two‐way ANOVA and Students′ *t* test was used to assess differences between the control and test groups. *P*‐values less than 0.05 were considered statistically significant.

## 3 RESULTS

### 3.1 MBG scaffolds containing Sr promotes periodontal regeneration whereas represses hnRNPL expression

**Figure 1:**
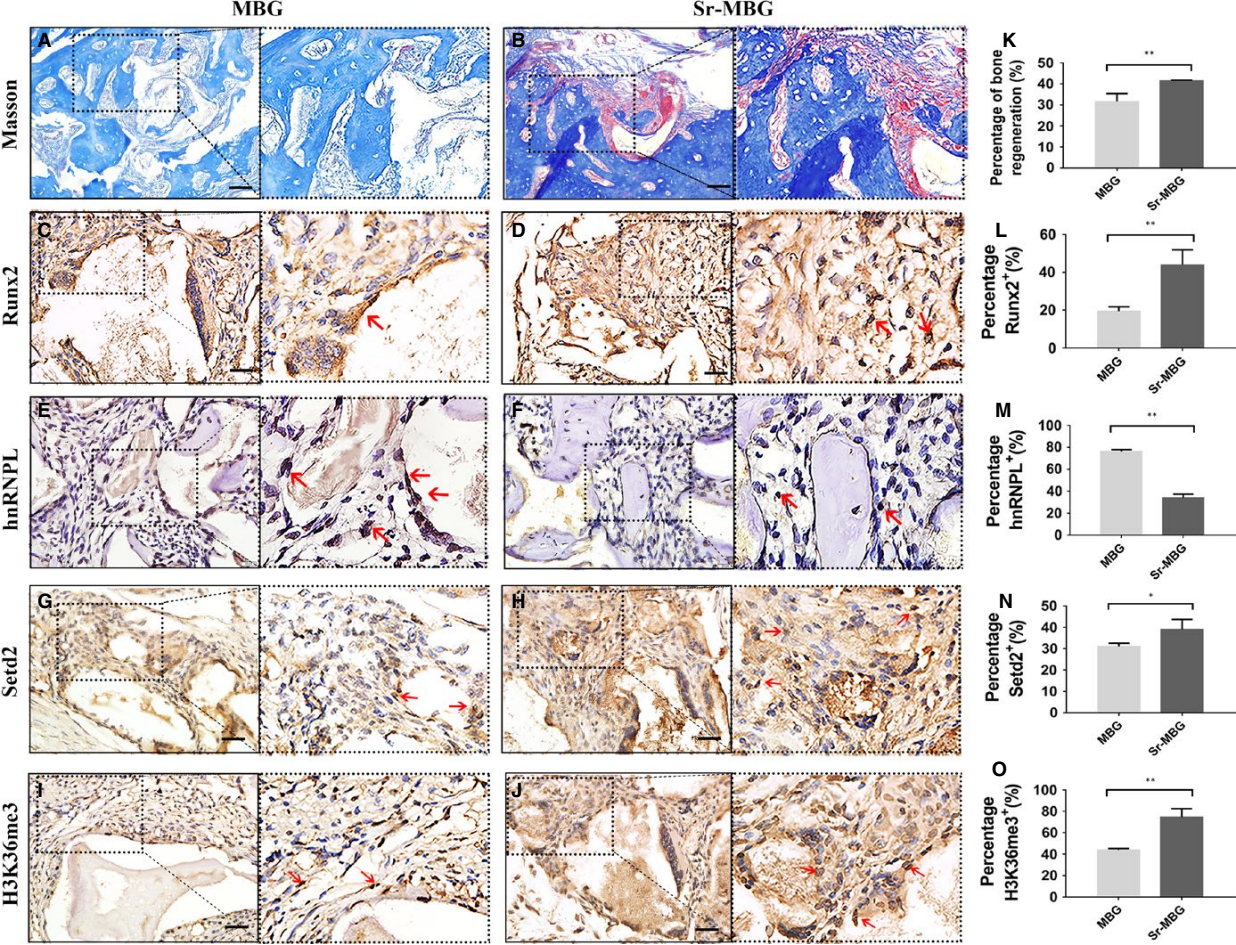
Regenerative potential and expression of hnRNPL, Setd2 and H3K36me3 in the healing of bone defects filled with MBG and Sr‐MBG. (A, B) Masson staining; (C‐J) immunohistochemistry staining with Runx2‐antibody (C, D), hnRNPL‐antibody (E, F), Setd2‐antibody (G, H) and H3K36me3‐antibody (I, J) in tissues from control and Sr‐MBG groups. scale bar = 20 μm; (K‐O) Quantitative analysis of new bone formation (K) and immuno‐histochemical staining of Runx2(L), hnRNPL (M), Setd2 (N) and H3K36me3 (O) positive cells between groups. *P ＜ 0.05; **P ＜ 0.01; ***P ＜ 0.001

Firstly, the effect of Sr‐MBG scaffolds on periodontal regeneration was investigated in periodontal fenestration defect of osteoporotic rats. Masson staining demonstrated that defects loaded with Sr‐MBG scaffolds had visibly more new bone formation and vascular distribution in the healing area than MBG scaffolds (Figure 1A,B,K). To investigate the osteogenic ability of Sr, immuno‐histochemical staining of Runx2, one of the early osteogenic markers, was performed. More frequent Runx2‐positive cells were detected in the presence of Sr (Figure 1C,D,L) while the percentage of hnRNPL‐positive cells was less in Sr‐MBG group (Figure 1E,F,M). This result implicated there may be some regulatory role of hnRNPL in the periodontal regeneration stimulated by Sr.

### 3.2 SrCl_2_ in the concentration of 1 mmol/L promotes PDLCs osteogenic differentiation without influencing proliferation

**Figure 2:**
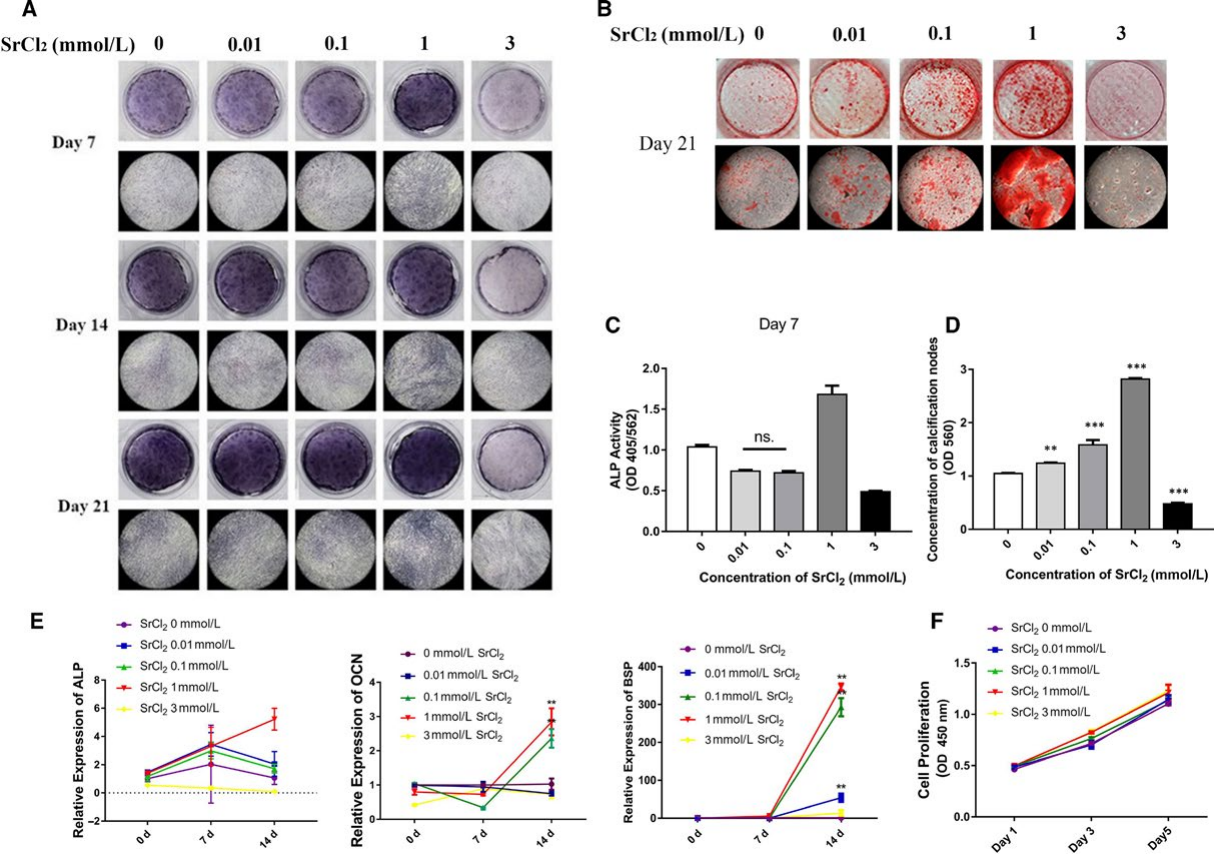
Role of various concentrations of SrCl_2_ (0, 0.01, 0.1, 1, 3 mmol/L) on PDLCs osteogenic differentiation. (A) ALP staining of PDLCs cultured in osteoblast differentiation media with or without SrCl_2 _at 7, 14 and 21 d. (B) Alizarin red staining of PDLCs at 21 d. (C, D) Quantification of ALP staining at 7 d (C) and alizarin red staining (D) of PDLCs stimulated by SrCl_2_ in different concentrations. (E)Relative expression of osteogenic differentiation markers of ALP, OCN and BSP of PDLCs stimulated by SrCl_2_ in different concentrations. (F) Cell proliferation of PDLCs assessed by CCK8 assay. *P ＜ 0.5; **P ＜ 0.01; ***P ＜ 0.001

We then investigated the mechanism of osteoblastic differentiation stimulated by Sr in vitro. To determine the optimal concentration of Sr, PDLCs were cultured in osteogenic differentiation media with SrCl_2 _at various concentrations ranging from 0 to 3 mmol/L. The results showed that the ALP activity in 0.01, 0.1 and 3 mmol/L groups were decreased after 7 days of induction (Figure 2A,C). After 14 days of induction, the expression levels of ALP in the three groups were also declined (Figure 2E) while the ALP activityand the expression levels of osteogenic markers such as ALP, OCN and BSP in 1 mmol/L group were all increased and highest among all groups (Figure 2C,E). It was also observed that the influence of SrCl_2 _to the calcification ability of PDLCs was dose‐dependent when the concentration was less than 1 mmol/L. However, if the concentration was 3 mmol/L, it showed a negative effect on the calcification ability of PDLCs (Figure 2B,D). Then we suspected if this effect was due to the proliferation of PDLCs, whereas the results showed no effect of the concentration of SrCl_2 _on the proliferation of PDLCs (Figure 2F).

### 3.3 SrCl_2_ promotes PDLCs osteogenic differentiation through AKT pathway

**Figure 3:**
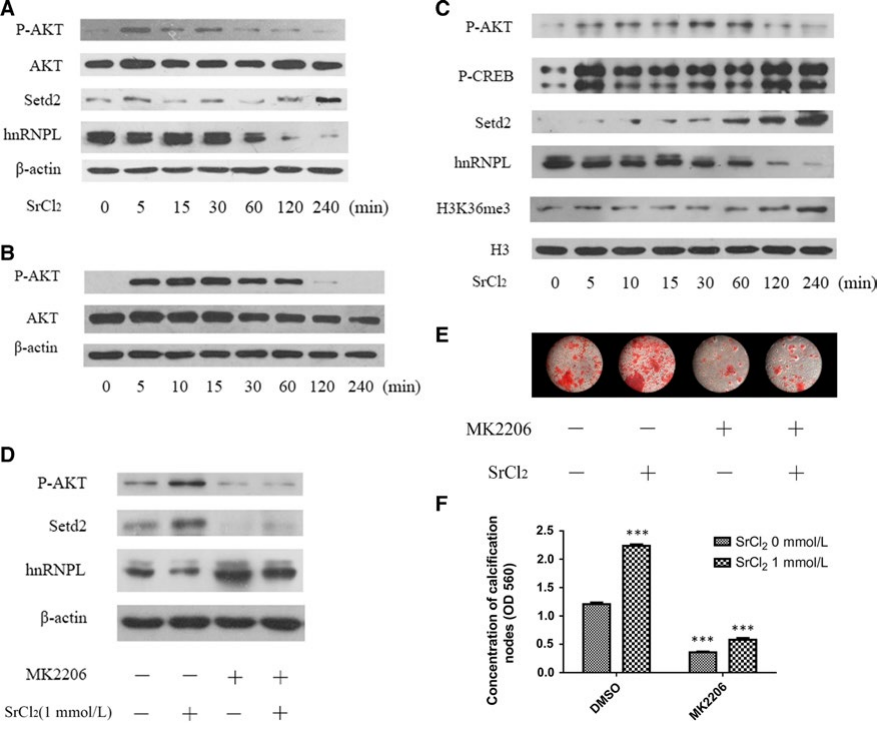
AKT pathway was activated by SrCl_2 _stimulation in PDLCs. (A‐C) PDLCs were first starved for 24 h, then stimulated by 1 mmol/L SrCl_2_ at different time periods ranging from 0 to 240 min. Then the total protein (A), cytoplasm protein (B) and nuclear protein (C) were extracted and immunoblotted using antibodies using AKT, hnRNPL and Setd2 antibody separately. (D) PDLCs were pretreated by MK2206 for 12 h to inhibit AKT signaling pathway, followed by stimulation with 1 mmol/L SrCl_2_. Total protein was collected and detected by immunoblot analysis of hnRNPL and Setd2. (E) PDLCs pretreated with MK2206 for 12 h were then induced towards osteogenic differentiation and alizarin red staining was performed on day 21. (F) Quantification of alizarin red staining. *P ＜ 0.5; **P ＜ 0.01; ***P ＜ 0.001

Strontium was shown to activate calcium sensing receptor (CaSR) and downstream protein phosphorylation and to promote osteogenesis.^9^ AKT is one of the most important protein kinases downstream of CaSR. Furthermore, AKT pathway was involved in Sr induced osteogenesis and angiogenesis.^27^ Therefore, we investigated the activity of AKT pathway in PDLCs stimulated by SrCl_2_ at various time points ranging from 15 minutes to 4 hours. The results showed that AKT pathway was activated by SrCl_2 _at 15 minutes and up to 1 hour both in the nucleus and cytoplasm (Figure 3A‐C). CREB, the downstream protein of the AKT pathway, was also phosphorylated at 15 minutes after SrCl_2_ stimulation (Figure 3C). Moreover, PDLCs pretreated by AKT specific inhibitors MK2206 failed to form similar mineralized nodes compared to the normal PDLCs with Sr stimulation (Figure 3D‐FC), indicated the positive role of AKT pathway in the differentiation process stimulated by Sr.

### 3.4 HnRNPL represses PDLCs osteogenic differentiation stimulated by Sr

Since hnRNPL was expressed in low levels in the Sr‐MBG group in vivo, we then examined the expression of hnRNPL in PDLCs. Western blot results showed hnRNPL was significantly decreased from 2 hours after SrCl_2_ stimulation both in total and nuclear protein (Figure 3A,C). With the pretreatment of AKT inhibitor MK2206, Sr failed to repress the expression of hnRNPL in PDLCs (Figure 3D), indicating that the repression of Sr to hnRNPL was AKT‐associated.

**Figure 4:**
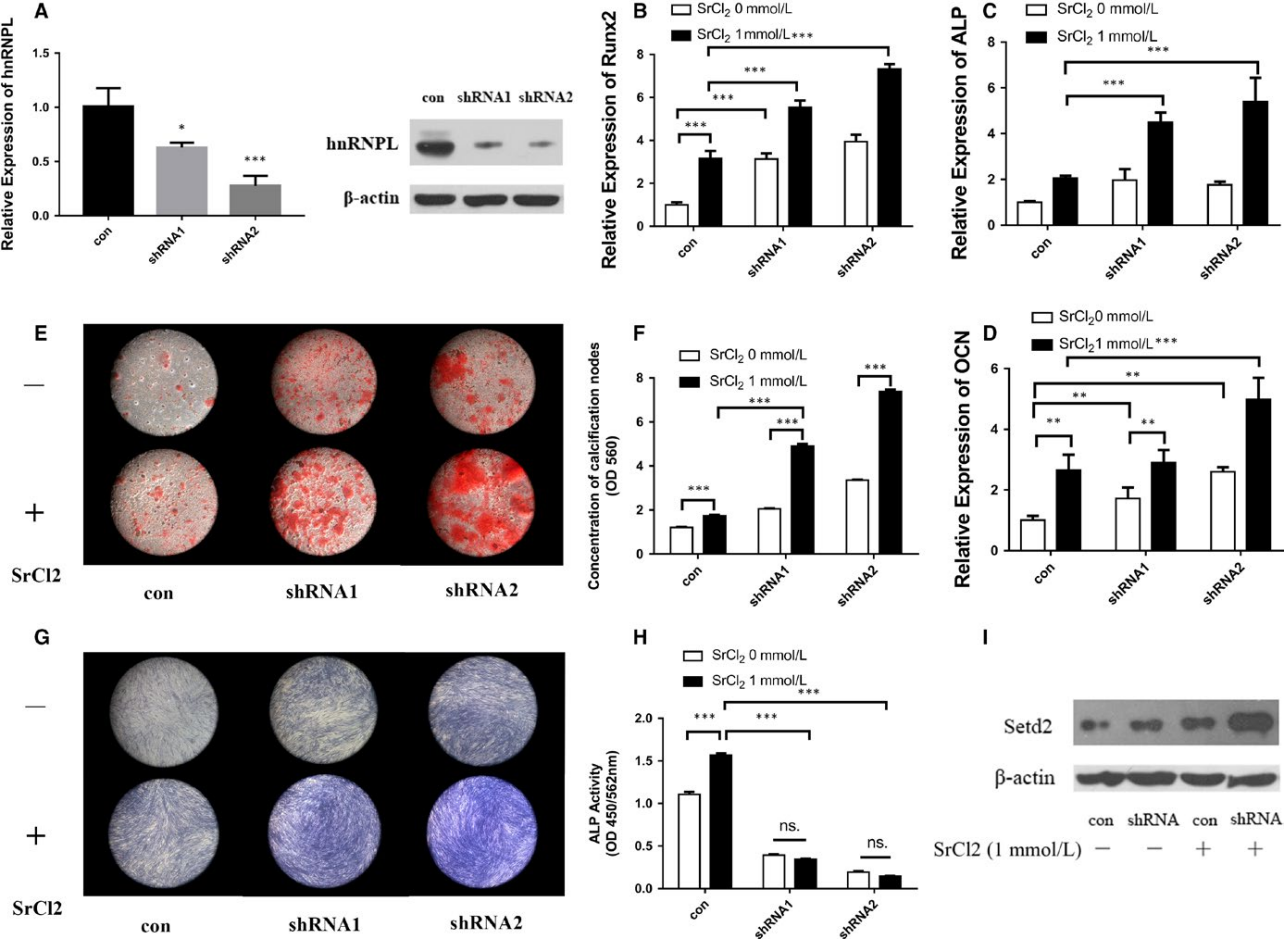
HnRNPL has negative effect on PDLCs osteogenic differentiation. (A) PDLCs were transfected by lentivirus to silence hnRNPL and real‐time PCR and western blot were utilized to detect the knock‐down proficiency of hnRNPL. (B‐D) Expression level of osteogenic genes, Runx2 (B), ALP (C), OCN (D) of PDLCs after hnRNPL knockdown and induction towards osteogenic differentiation with or without SrCl_2 _stimulation. Alizarin red staining (E) and quantification of mineralization (F), ALP staining (G) and quantification of ALP activity (H) of PDLCs transfected by lentivirus with scrambled and hnRNPL‐shRNA vectors after osteogenic induction with or without SrCl_2_. (I) Expression level of Setd2 of PDLCs after hnRNPL knockdown and induction towards osteogenic differentiation with or without SrCl_2 _stimulation. *P ＜ 0.5; **P ＜ 0.01; ***P ＜ 0.001

To further investigate the role of hnRNPL in the process of PDLCs osteogenic differentiation stimulated by Sr, we silenced hnRNPL in PDLCs with two shRNAs (Figure 4A). It was found that mineralization, ALP activity and the expression levels of osteogenic‐related markers ALP, Runx2 and OCN were all increased after interfering the expression of hnRNPL with or without Sr stimulation (Figure 4B‐H).

### 3.5 HnRNPL can inhibit the expression of Setd2 to suppress PDLCs osteogenic differentiation stimulated by Sr

Setd2‐mediated H3K36me3 has also previously been shown to be involved in the regulation of alternative splicing,^28^ but whether it is associated with the splicing process mediated by hnRNPL is remained unknown. Immunohistochemistry results showed that the percentages of Setd2‐positive and H3K36me3‐positive cells are significantly higher in the group containing Sr in vivo (Figure 1G‐O). It was revealed that the expression of Setd2 was significantly increased from 2 hours after SrCl_2_ stimulation (time when hnRNPL decreased). When pre‐treated with the AKT inhibitor MK2206, the expression of Setd2 failed to be promoted by Sr in PDLCs (Figure 3D). More importantly, when hnRNPL was silenced, the expression of Setd2 was significantly increased in PDLCs, with the highest expression observed following Sr stimulation (Figure 4I). These results implied that the AKT pathway promoted Setd2 expression through repressing downstream hnRNPL.

**Figure 5:**
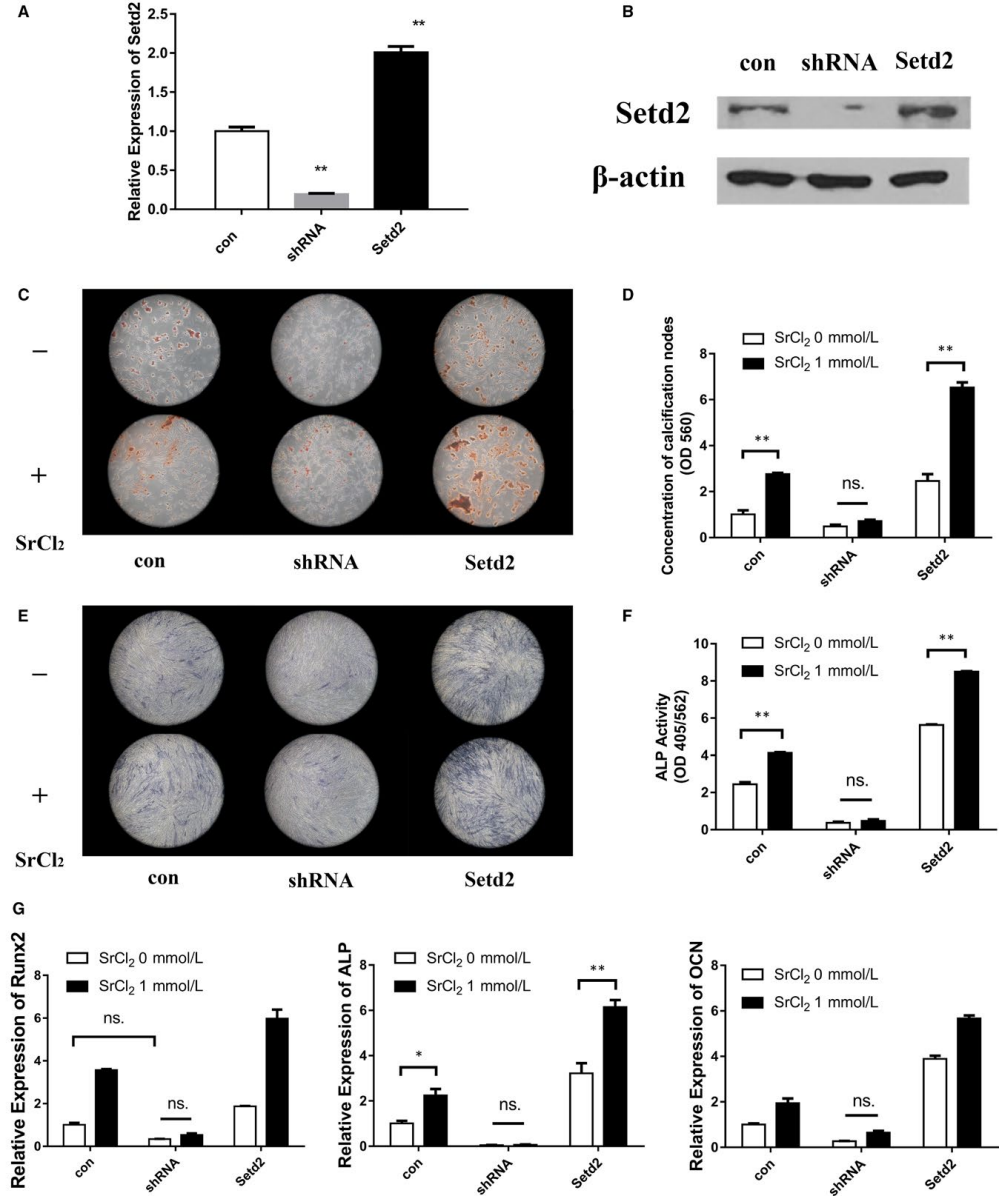
Setd2 promotes PDLCs osteogenic differentiation stimulated by Sr. PDLCs were transfected by lentivirus to knockdown or over‐express Setd2, with satisfactory expressions confirmed by real‐time PCR (A) and western blot (B) analysis. Alizarin red staining (C) and quantification of mineralization (D) of PDLCs transfected by lentivirus with scrambled, Setd2‐shRNA and Setd2‐overexpressing vectors following osteogenic differentiation with or without SrCl_2_. ALP staining (E) and quantification of ALP activity (F) of PDLCs transfected by lentivirus with scrambled, Setd2‐shRNA and Setd2‐overexpressing vectors following osteogenic differentiation with or without SrCl_2_. (G) Expression level of osteogenic genes, Runx2, ALP, OCN of PDLCs after Setd2 knockdown or over‐expression and induction towards osteogenic differentiation with or without SrCl_2 _stimulation. *P ＜ 0.5; **P ＜ 0.01; ***P ＜ 0.001

However, whether Setd2 played a positive role similar to the AKT pathway remained unknown. We therefore investigated the role of Setd2 during Sr‐induced osteogenic differentiation of PDLCs by Setd2 knock‐down or over‐expression (Figure 5A,B). The results showed that mineralization, ALP activity and the expression of osteogenic‐related markers ALP, Runx2 and OCN were decreased in the knock‐down group in PDLCs even when stimulated with SrCl_2_ (Figure 5C‐G). In contrast, over‐expression of Setd2 in PDLCs showed that SrCl_2_ can further enhance osteogenic differentiation (Figure 5C‐G).

## 4 DISCUSSION

Periodontal tissue regeneration has been a major problem for clinicians for a long time, of which periodontal bone regeneration is the most important part. Periodontal tissues include gingiva, cementum, periodontium and alveolar bone. Osteoporosis is a disease characterized by reduced bone density, causes more complex treatment and poor prognosis of periodontal defects.^29^ The molecular mechanisms underlying this bone condition are related to estrogen deficiency and overproduction of some cytokines.^30^ Strontium Ranelate is believed to be effective in osteoporosis treatment, and many studies have confirmed that strontium ions can prevent osteoporosis by regulating osteoblasts and osteoclasts. In the treatment of periodontal tissue destruction, there is a need to discover key factors of bone metabolism as potential targets for the treatment and management osteoporosis.

Mesoporous bioactive glass (MBG) with well‐ordered mesoporous channels has been shown to facilitate osteogenesis and bioactivity in vivo.^5^, ^6^, ^7^, ^8^, ^31^ In addition, the ions and degradation products of MBG have been reported to effectively promote the expression of osteogenic genes and proteins. Various groups have now combined biomaterials with strontium (Sr) ions in order to improve their biological activity and osteogenic potential.^14^, ^32^ Our research group recently demonstrated that the MBG scaffolds combined with Sr, efficiently promoted periodontal regeneration in rat periodontal fenestration defects even in severely osteoporotic animals.^10^ In the present study, immunohistochemistry staining of Runx2 was performed to detect the early osteogenic differentiation ability of PDLCs around these biomaterials. The results showed that the larger proportion of Runx2‐positive cells was detected in the Sr‐MBG group and Masson staining also showed more bone formation in the Sr‐MBG group.

**Figure 6:**
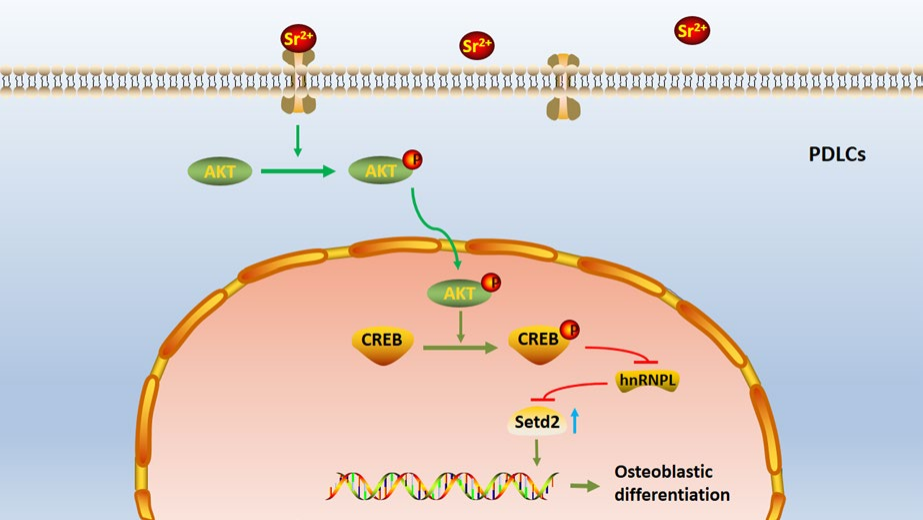
Schematic diagram of the mechanism involved in Sr promoted‐PDLCs osteogenic differentiation

Strontium was previously shown to activate calcium sensing receptor (CaSR) and downstream protein phosphorylation to promote osteogenesis^9^. Activated CaSR can promote the activation of phosphatidyl inositol ‐3‐ hydroxylase (PI3K), sequentially catalyse substrate diphosphoinositol to produce triphosphoinositol. Triphosphoinositol can following activate AKT through promoting phosphoinositol depending protein kinase (PDK) activation.^33^ To investigate the mechanism of the effect of Sr on PDLCs, we examined one of the classical protein phosphorylation pathway, AKT pathway. As expected, Sr promoted PDLCs osteogenic differentiation through activation of AKT pathway (Figure 6). AKT is a kind of serine/threonine protease containing PH domain, which can combine with phosphatidyl inositol triphosphate (PIP3) on the inner side of cell membrane. After activated by PDK, AKT leaves the cell membrane and enters the nucleus, then promotes the expression of downstream genes.^34^ Therefore, we tested the expression levels of p‐AKT in cytoplasm protein and nucleus protein. The results showed the increased nuclear import of AKT and phosphorylated CREB, which is the downstream transcription factor of AKT. Furthermore, by blocking the AKT pathway，we verified that hPDLCs osteogenic differentiation promoted by SrCl_2_ is indeed AKT‐dependent.

Some previous studies demonstrated that several hnRNP family members changed significantly in BMSCs during osteogenesis and can regulate gene transcription and RNA splicing in bone metabolism.^35^, ^36^ The change of hnRNPL expression after drug treatment in osteosarcoma cells indicates that hnRNPL was involved in the physiological activity of bone‐related cells,^37^ and the alternative splicing mediated by hnRNPL is associated with protein phosphorylation.^38^ We therefore investigated the expression and effect of hnRNPL in PDLCs during the osteogenic differentiation with Sr stimulation. The results showed a negative role of hnRNPL in this process and also suppressed by AKT pathway.

Recently one study showed histone H3K36 methylation affected alternative splicing in plants with H3K36me3 being involved in the regulation of alternative splicing.^28^ The methylation of H3K36 was also proposed to be associated with osteogenesis.^39^, ^40^ We then detected Setd2, an H3K36me3‐specific histone methyltransferase, in the process of PDLCs osteogenesis. The results showed a significant increase of Setd2 expression in PDLCs stimulated by Sr in vivo and in vitro. This increase is achieved by suppression of hnRNPL. Apart from this, a positive role of Setd2 was observed during periodontal regeneration stimulated by Sr.

We hypothesis there may be several reasons of the effect of hnRNPL: 1. HnRNPL may cause some related factors to the pre‐mRNA of Setd2, and cause stranded local introns and decreased expression of Setd2; 2. HnRNPL may reduce the production of other factors that promote the expression of Setd2 by giving rise to abnormal splicing of these proteins; 3. HnRNPL may inhibit the expression of Setd2 by increasing the production and recruitment of inhibiting or repressor proteins to suppress the expression of Setd2.

In conclusion, we provide rational describing the role of hnRNPL and Setd2 during periodontal regeneration. It is therefore possible to target hnRNPL and Setd2 as potential novel therapeutic targets for the treatment of periodontal tissue destruction most notably in osteoporotic patients.

## CONFLICT OF INTEREST

The authors confirm that there are no conflicts of interest.

## AUTHOR CONTRIBUTION

Study design: RJM, MW, RJ, YZ and YL. Study conduct: XJ. Data collection: XJ and CY. Data analysis: XJ, HX and JW. Data interpretation: XJ, RJM, TL, YZ and YL. Drafting manuscript: XJ, RJM and TL. Revising manuscript content: all authors. Approving final version of manuscript: all authors.
